# Genomic characterization of metastatic ultra-hypermutated interdigitating dendritic cell sarcoma through rapid research autopsy

**DOI:** 10.18632/oncotarget.26352

**Published:** 2019-01-08

**Authors:** Hui-Zi Chen, Russell Bonneville, Lianbo Yu, Michele R. Wing, Julie W. Reeser, Melanie A. Krook, Jharna Miya, Eric Samorodnitsky, Amy Smith, Dorrelyn Martin, Thuy Dao, Qishan Guo, David Liebner, Aharon G. Freud, Patricia Allenby, Sameek Roychowdhury

**Affiliations:** ^1^ Department of Internal Medicine, Division of Medical Oncology, Comprehensive Cancer Center, The Ohio State University, Columbus, Ohio, USA; ^2^ Department of Internal Medicine, Hematology and Oncology Fellowship Program, Comprehensive Cancer Center, The Ohio State University, Columbus, Ohio, USA; ^3^ Biomedical Sciences Graduate Program, The Ohio State University, Columbus, Ohio, USA; ^4^ Department of Biomedical Informatics, The Ohio State University, Columbus, Ohio, USA; ^5^ Department of Pathology, Division of Hematopathology, The Ohio State University, Columbus, Ohio, USA; ^6^ Department of Pathology, Division of Autopsy Services, The Ohio State University, Columbus, Ohio, USA

**Keywords:** tumor heterogeneity, research autopsy, hypermutation

## Abstract

Interdigitating dendritic cell sarcoma (IDCS) is an extremely rare cancer of dendritic cell origin that lacks a standardized treatment approach. Here, we performed genomic characterization of metastatic IDCS through whole exome sequencing (WES) of tumor tissues procured from a patient who underwent research autopsy. WES was also performed on a treatment-naïve tumor biopsy sample obtained from prior surgical resection. Our analyses revealed ultra-hypermutation, defined as >100 mutations per megabase, in this patient's cancer, which was further characterized by the presence of three distinct mutational signatures including UV radiation and APOBEC signatures. To characterize clonal heterogeneity, we used the bioinformatics tool Canopy to leverage single nucleotide and copy number variants to catalog six subclones across various metastatic tumors. Truncal alterations, defined as being present in all clonal tumor cell populations, in this patient's cancer include point mutations in *TP53* and *CDKN2A* and amplifications of *c-KIT* and *APOBEC3A-H*, which are likely driver mutations. In summary, we have performed genomic characterization evaluating tumor mutational burden (TMB) and heterogeneity in a patient with metastatic IDCS. Despite ultra-hypermutation, this patient's cancer was not responsive to treatment with PD-1 inhibition. Our results underscore the importance of characterizing clonal heterogeneity in TMB-high cancers.

## INTRODUCTION

Interdigitating dendritic cell sarcoma (IDCS) is an extremely rare malignancy of dendritic cell origin with approximately 100 cases reported to date [1, 2]. Due to its rarity and challenging diagnosis, genomic characterization of this neoplasm has not been previously reported. Furthermore, no standard therapy exists for IDCS, which tends to affect middle-age adults with median age of diagnosis of 56.5 years [3]. Localized IDCS constitutes 47% of cases and manifests as painless lymphadenopathy, most commonly involving the cervical and axillary nodes. Isolated extra-nodal disease occurs in 25% of cases, involving the liver, lung, spleen, bone marrow and gastrointestinal tract [3]. Distant metastases occur in 39% of cases and most frequently involved lymph nodes, lung, liver and bone marrow [3].

Here we performed whole exome sequencing (WES) of multiple tumors obtained through rapid research autopsy of a patient with metastatic IDCS. To our knowledge, this is the first time IDCS has been extensively sequenced and analyzed. Our findings demonstrate a rare ultra-hypermutated genotype, with >100 somatic mutations per megabase of genome (mut/Mb) [4], characterized by distinct mutational signature profiles and clonal diversity that occurred early in the evolution of this patient’s cancer. Phylogenetic analysis revealed inactivation of *TP53* and *CDKN2A* (*p16*^*INK4a*^*/p19*^*ARF*^) as well as amplification of *c-KIT* and *PDGFRα* as truncal alterations. We further detected amplification of *APOBEC3A-H* encoding cytidine deaminases on chromosome 22q that may have attributed to the ultra-hypermutation. In summary, our work describes the genomic landscape and clonal heterogeneity of an ultra-rare cancer through the innovative approach of research autopsy.

### Diagnosis and treatment

**Figure 1:**
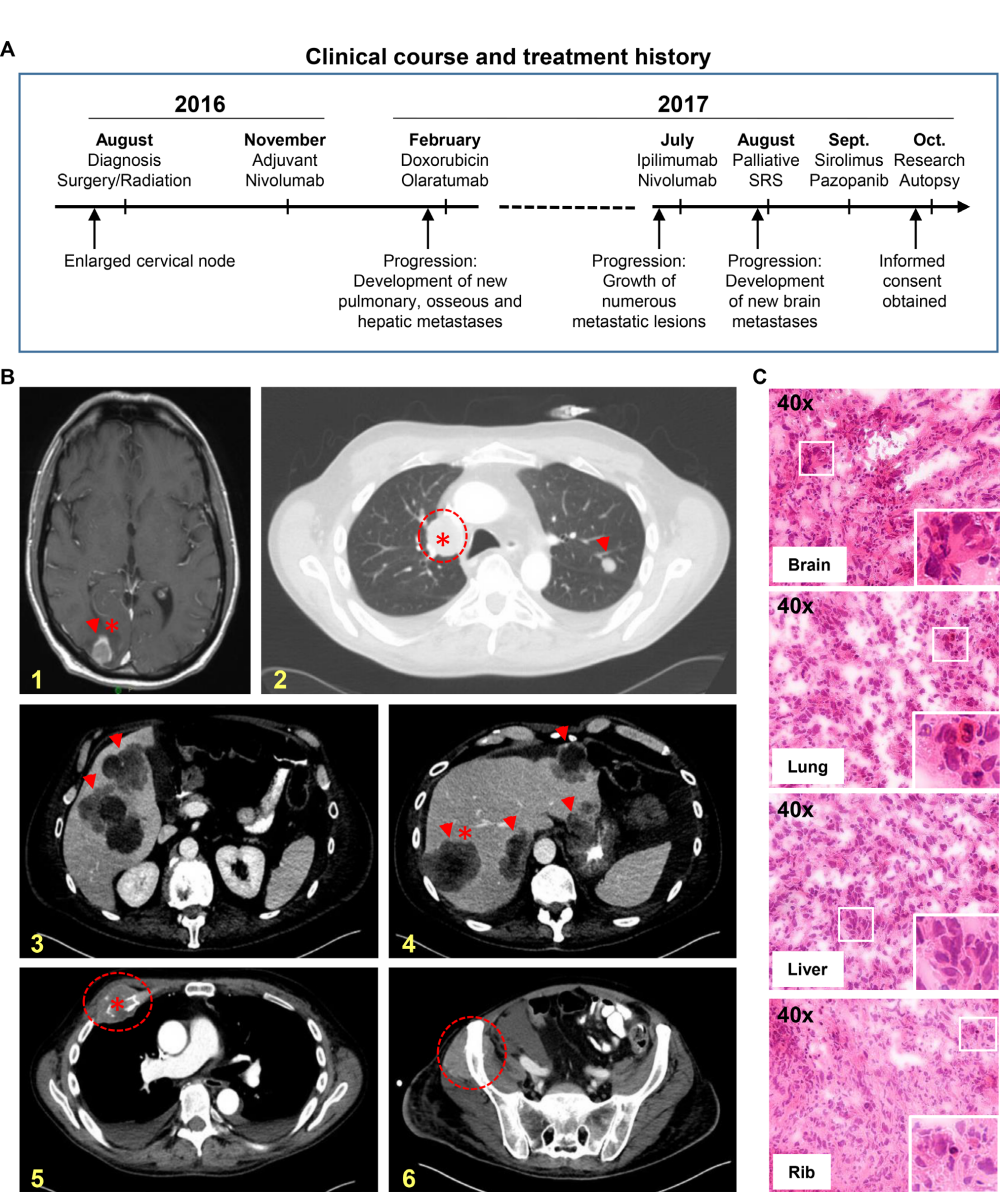
Research autopsy of a patient with metastatic IDCS (**A**) Summary of clinical course and treatment history of the patient. Research autopsy was performed 3 hours post-mortem. SRS, stereotactic radiosurgery. (**B**) CT scans depicting organs with metastatic cancer; brain (panel 1, yellow), hilar lymph node (2), liver (3-4), rib (5) and pelvis (6). Arrowheads and dashed circles indicate tumors; asterisks indicate tumors procured at research autopsy. (**C**) H&E stained frozen sections of metastatic tumors in brain, lung, liver and rib procured from research autopsy. Sections demonstrate high tumor cell content. Enlarged inset at bottom right of each image panel shows dysplastic nuclei with mitotic figures.

Our patient was a 57-year-old Caucasian male who developed an isolated FDG-avid 2.4 × 2.4 cm right-sided neck mass on PET scan in 2016. Biopsy result of this mass showed a “pleomorphic/spindle cell neoplasm”. Immunohistochemical (IHC) analyses demonstrated focal CK7 staining. Clinical evaluation revealed no primary lesion involving the skin or oropharynx. In August 2016, he underwent modified radical right neck dissection and partial submandibular gland excision with one level I lymph node demonstrating complete tumor involvement and suspicious for extranodal extension; remaining lymph nodes (0/29) from levels II, III and IV were negative for tumor infiltration. IHC on the surgical specimen showed positivity for S100, SOX10 and CK7. A diagnosis of IDCS was issued. Given his localized presentation, he received adjuvant radiation to the right level IB-III nodes with a boost to the primary site of disease. In November 2016, he initiated adjuvant nivolumab (3 mg/kg every 2 weeks) given FoundationOne^®^ report showing >100 mut/Mb in his tumor. Following adjuvant immunotherapy, he developed metastatic recurrence that failed to respond to subsequent treatments including combination immunotherapy (CTLA-4/PD-1 inhibitors), chemotherapy and molecularly targeted therapies (Figure 1). His clinical course leading up to the research autopsy is summarized in Figure 1A.

## RESULTS

### Rapid research autopsy

**Figure 2:**
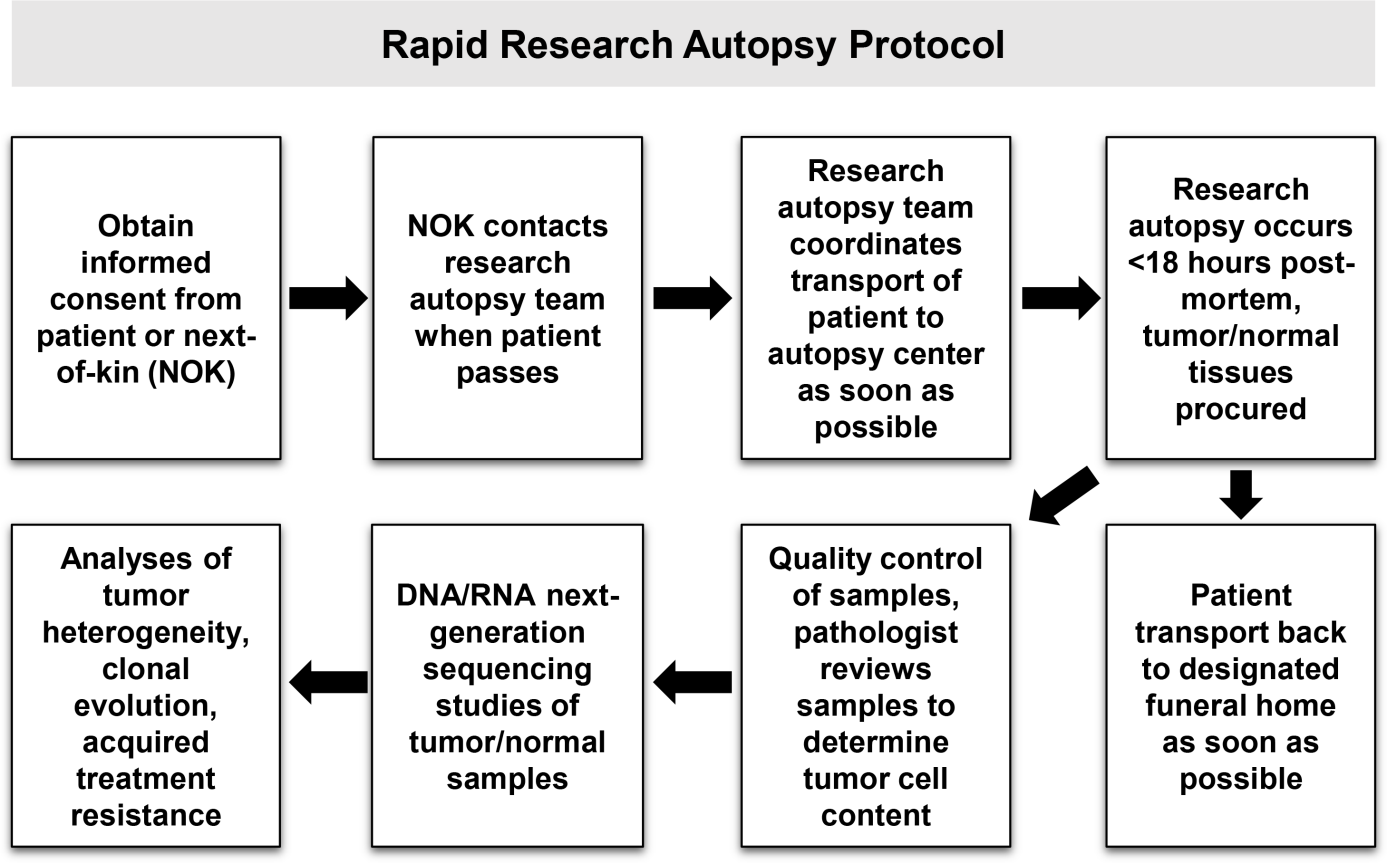
Overview of research autopsy protocol

Prior to his death, the patient was consented to an Institutional Review Board (IRB)-approved research autopsy study for patients with advanced cancers (Figure 2). CT scans obtained prior to hospice enrollment showed tumor involvement of multiple organs (Figure 1B) and were used to guide sample procurement at time of research autopsy, which was performed within three hours post-mortem. A total of twenty-four metastatic tumor samples were procured from involved organ sites (Figure 1B, ^*^tumors). A pathologist assessed the viability and tumor cell (TC) content of autopsy samples (Figure 1C) prior to selection for genomic studies.

### Genomic characterization of IDCS

**Figure 3:**
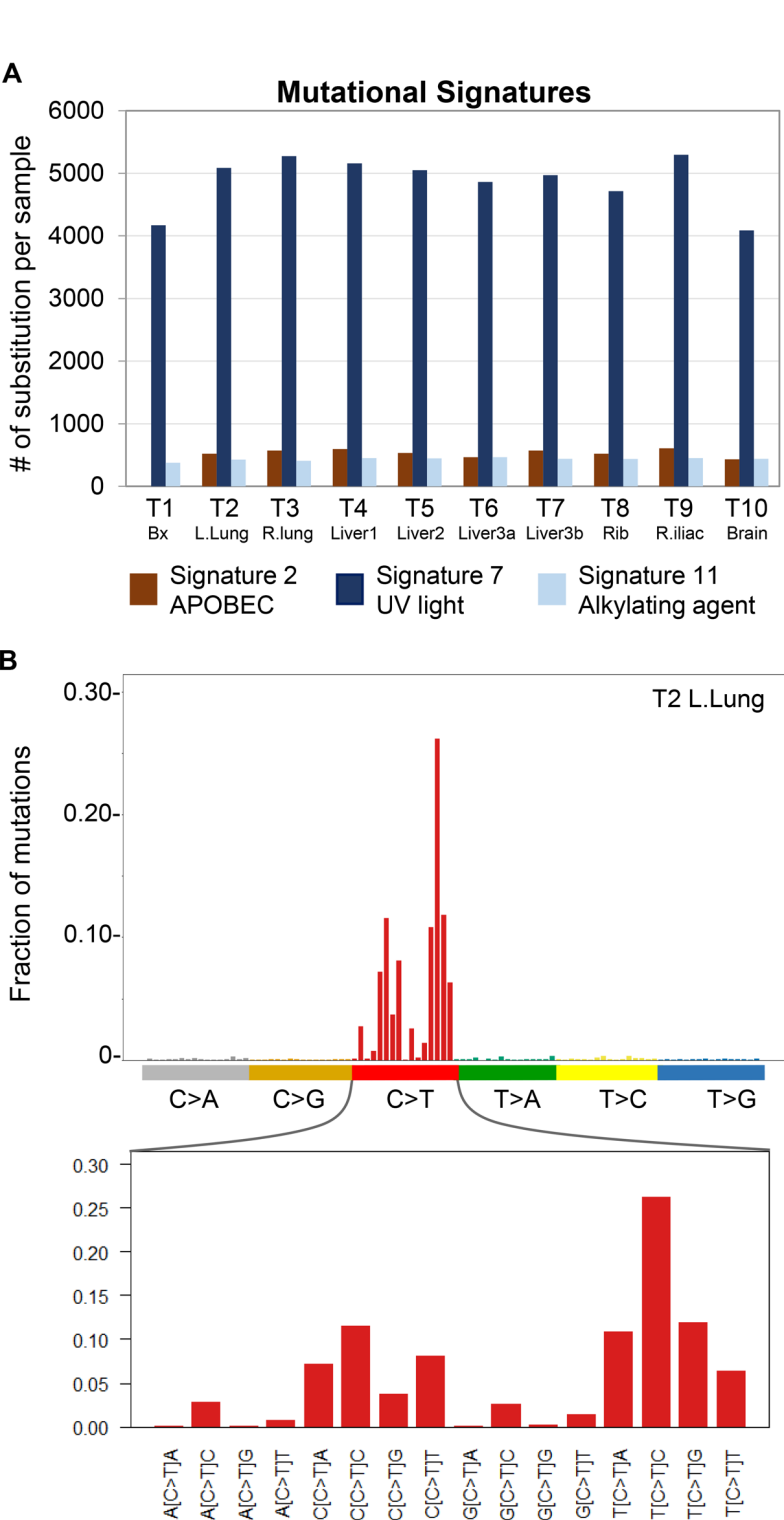
Detection of mutational signature profiles in metastatic IDCS (**A**) Mutational signatures in tumor samples were called with deconstructSigs. Among the thirty signatures in the COSMIC Mutational Signatures set, only Signatures 2 (APOBEC), 7 (UV) and 11 (alkylating agents) were detected. T1-10 are as labeled and correspond to samples listed in Table 1. (**B**) Representative lego plot of a sequenced tumor (T2) demonstrating the predominance of C>T transitions in the three signatures detected in this patient’s cancer. The X-axis of lego plots contains 96 possible mutation types that result when the six classes of base substitutions (e.g. C>T) are placed in the trinucleotide context of flanking 5′ and 3′ bases. The Y-axis represents the fraction of base substitutions in sample T2 within each trinucleotide context.

**Figure 4:**
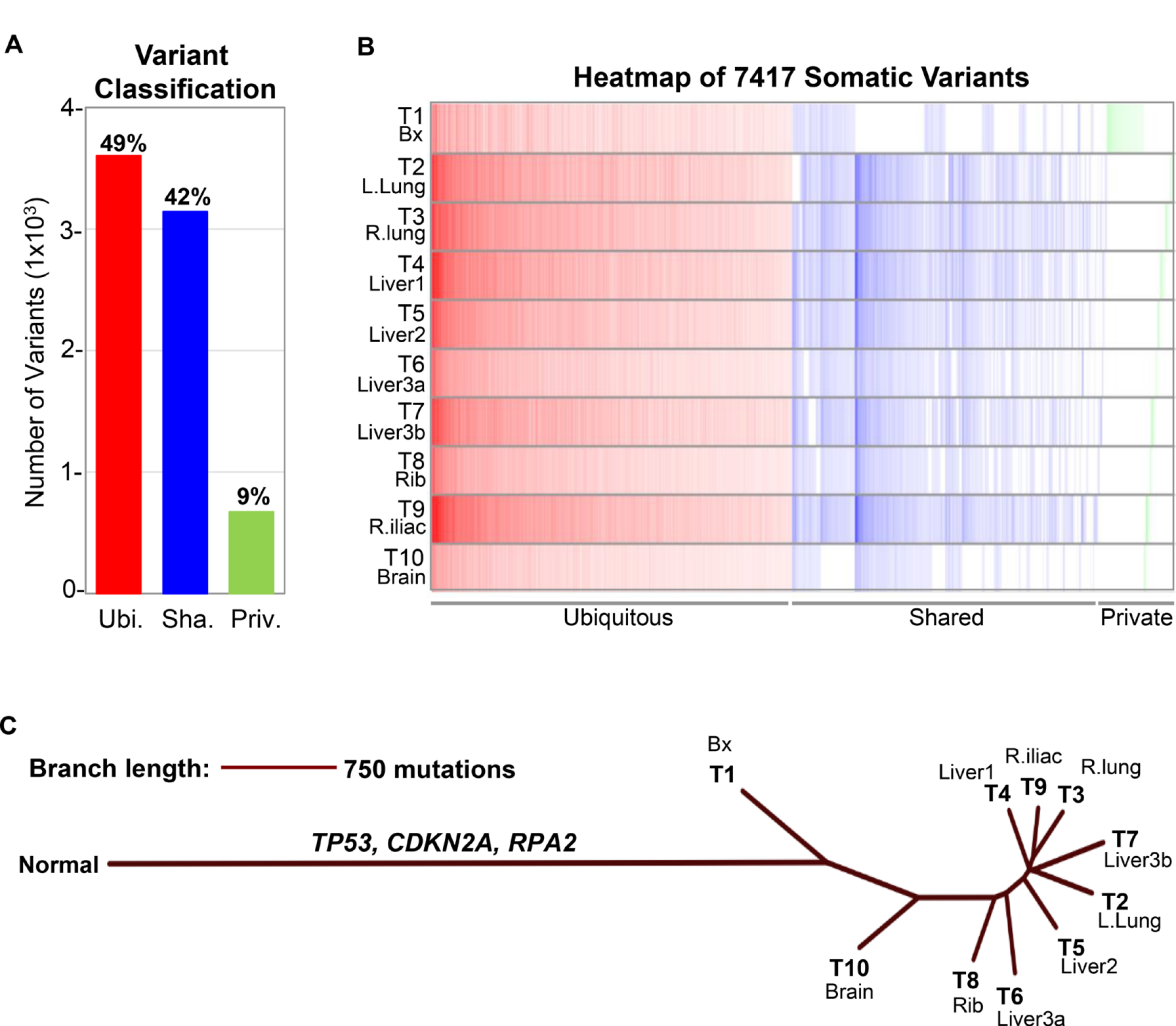
Classification of somatic variants and construction of neighbor joining (NJ) tree (**A**) The 7,417 somatic variants detected in all ten tumor samples T1-10 from this patient were classified into three categories: Ubiquitous (Ubi.), present in all ten tumor samples; Shared (Sha.), present in some but not all ten tumor samples; Private (Priv.), present in only one tumor sample. Percentage of each variant category are as indicated in the bar graph. Somatic variants analyzed include single nucleotide variants (SNVs) and insertions/deletions (indels). (**B**) Heatmap demonstrating distribution and variant allele frequency (VAF) of the 7,417 somatic variants in ten tumor samples T1-10. Color intensity corresponds to VAF. (**C**) NJ tree depicting the evolutionary relationship of the ten tumor samples T1-10 from this patient. Normal represents hypothetical population of wildtype cells without somatic aberrations. The 3,642 ubiquitous variants included driver mutations *TP53* and *CDKN2A* as well as the *RPA2* variant predicted to have loss-of-function. Branch lengths correspond to the genetic distance between samples. T1-10 corresponds to samples listed in Table 1.

We performed WES on nine metastatic tumors and the resected pre-treatment tumor (Table 1). Calculation of tumor mutational burden (TMB), including single nucleotide variants (SNVs) and insertions/deletions (indels), demonstrated ultra-hypermutation (130.1–167.0 mut/Mb) in all tumors analyzed (Table 1; Supplementary Table 1). Interrogation of microsatellite status utilizing MANTIS [5] showed that all tumors were microsatellite stable (MSS) despite their ultra-hypermutation (Table 1). To further characterize this patient’s cancer, we investigated whether specific substitution mutational signatures, which arise due to different mutagenic processes [6], may be present. This analysis revealed the presence of Signature 2 (APOBEC), Signature 7 (UV light) and Signature 11 (alkylating agent), which are all characterized by C>T substitutions, in all tumor samples (Figure 3A–3B; Supplementary Figure 1 and Supplementary Table 2). Of the three mutational signatures, Signature 7 had the highest prevalence (Figure 3A). We next classified the 7,417 somatic variants (SNVs and indels) in all ten tumor samples of this patient into three categories: Ubiquitous, Shared and Private (Figure 4A–4B). Ubiquitous variants are variants detected in all tumor samples and included driver mutations in *TP53* and *CDKN2A*, while shared variants are those detected in subsets of tumor samples. Finally, private variants are variants unique to each tumor sample. A tumor-centric evolutionary tree was constructed based on the pairwise genetic distance, as measured by number of discordant SNVs, between the wildtype cells (normal) and tumor samples in this patient (Figure 4C). Finally, we interrogated the prevalence of copy number variations (CNVs) using the allele-specific CNV caller FALCON [7]. After manual review and curation, 29 unique CNVs including amplification (including *c-KIT*, *PDGFRα*, and *APOBEC3A-H*), deletion and loss-of-heterozygosity events were detected (Supplementary Figure 2 and Supplementary Tables 3–4).

**Table 1:** Sample characteristics and sequencing metrics

Sample	Organ involved	% Tumor cells	Median Coverage	Percent 100X	TMB (mut/Mb)	# SNVs	# Indels	Microsatellite
Normal	Blood	N/A	265	88.01	N/A	N/A	N/A	N/A
Tumor 1 (T1)	Biopsy (Bx)	30%	100	50.10	131.5	5,091	21	0.326, MSS
T2	Left lung (L.lung)	80%	213	79.83	158.4	6,139	22	0.330, MSS
T3	Right lung (R.lung)	90%	245	84.19	165.3	6,408	19	0.330, MSS
T4	Liver1	90%	218	82.12	163.3	6,329	20	0.328, MSS
T5	Liver2	80%	233	83.38	158.8	6,160	15	0.327, MSS
T6	Liver3a	80%	270	87.01	150.4	5,826	22	0.318, MSS
T7	Liver3b	90%	286	87.88	156.1	6,050	19	0.324, MSS
T8	Rib	80%	272	86.70	148.9	5,776	15	0.319, MSS
T9	Right iliac (R.iliac)	90%	242	85.20	167.0	6,473	20	0.330, MSS
T10	Brain	50%	257	86.80	130.1	5,043	15	0.320, MSS

A board-certified pathologist estimated the percentage of tumor cells in each sample. Liver3a and Liver3b samples were procured from two different regions of a large liver tumor. The pre-treatment biopsy sample was obtained from a formalin-fixed paraffin-embedded surgical specimen, while autopsy tumor samples were frozen in OCT. Percentage 100X indicates the percent of variants in each tumor sample with at least 100X coverage. TMB includes synonymous and non-synonymous SNVs as well as insertions/deletions (indels). #SNVs and #Indels columns indicate the total number of somatic SNVs and indels detected in each tumor sample. Microsatellite status was determined using MANTIS, with scores <0.4 indicating microsatellite stability (MSS).

### Reconstructing clonal evolution

**Figure 5:**
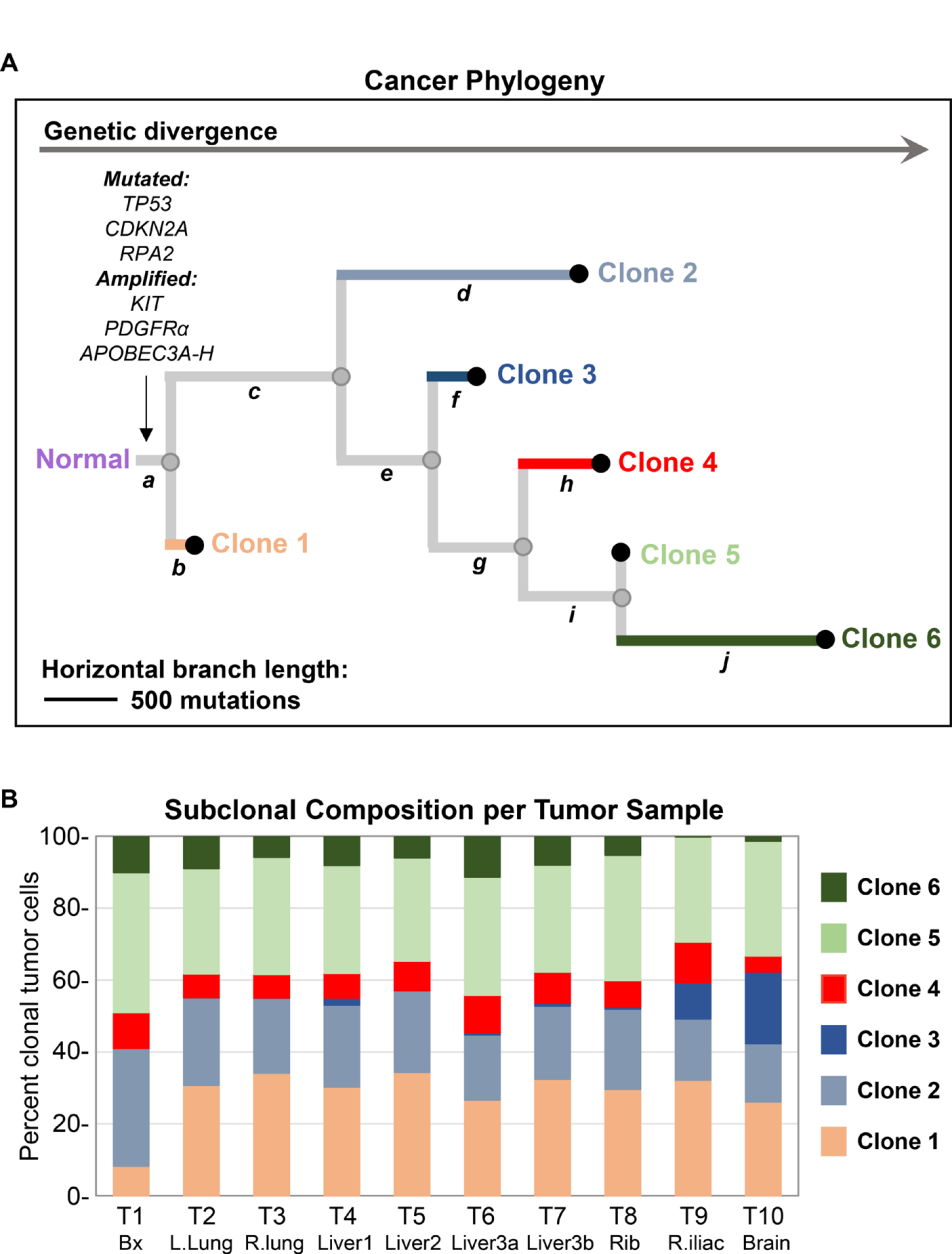
Analysis of cancer phylogeny and clonal evolution (**A**) Phylogram constructed using Canopy integrating SNVs, curated copy number variations (CNVs) and indels detected through WES of tumor samples T1-10. Output from Canopy includes number of clones, clonal fractions and mutational groups (a-j) associated with each clone. Given the large number of somatic SNVs in this ultra-hypermutated cancer, downsampling of SNVs was performed in addition to following recommended parameters for Canopy tree building. Somatic SNVs not utilized by Canopy as well as indels were retroactively assigned based on VAF patterns to mutational groups a-j using a maximal likelihood model. Based on this analysis, six different clones of tumor cells were identified in this patient’s cancer. Truncal or group a mutations are common or ancestral to all clones of tumor cells. In contrast, mutations in groups b, d, f, h, and ***j*** are private to clones 1, 2, 3, 4, and 6, respectively. The length of horizontal branches in the tree is proportional to the number of mutations. (**B**) Stacked bar graph depicting the percentage of different clonal tumor cell populations as estimated by Canopy in each sample. T1-10 corresponds to samples listed in Table 1.

Integrating the above genomic data (SNVs, indels and CNVs), we used the program Canopy [8] to synthesize a hypothetical model of the clonal evolution of this patient’s cancer (Figure 5A). Canopy identified six genetically distinct clonal populations of tumor cells, present in various proportions within each tumor (Figure 5B; Supplementary Table 5). These six clones are differentiated by ten unique groups of genomic alterations (Figure 5A, a–j; Supplementary Tables 6–7), which all contained mutational Signature 7 (Supplementary Figure 3 and Supplementary Table 2). Of note, group *a* contains truncal alterations ancestral to all tumor cells including two well-documented driver mutations, *TP53* P278L and *CDKN2A* R80X, which were classified as ubiquitous (Figure 4C). Interestingly, we also detected a truncal variant *RPA2* V108L. *RPA2* encodes a subunit of the heterotrimeric Replication Protein A complex that is critical for DNA replication, repair, recombination and DNA damage response [9]. Finally, we identified two notable truncal amplifications of regions on the long arms of chromosome 4 (∼17.2Mb), containing *c-KIT* and *PDGFRα*, and chromosome 22 (∼24.6Mb), containing *APOBEC3A-H* (Figure 5A; Supplementary Figure 2).

## DISCUSSION

Extensive multi-regional sequencing of tumors revealed the complex genetic landscape of treatment-refractory cancers by demonstrating intratumor heterogeneity [10, 11], which underscores the limitations of tumor profiling with tissue derived from a single biopsy specimen. From a clinical perspective, tumor heterogeneity contributes to the incomplete therapeutic responses seen in patients receiving different types of anti-cancer therapies including molecularly targeted therapies. Mechanistically, tumor heterogeneity drives acquired therapeutic resistance by facilitating the selection and expansion of therapy-resistant clones. Research autopsy has emerged as a powerful approach to characterize tumor heterogeneity at different metastatic sites in the individual patient. Together with information from treatment-naïve samples, sequencing of metastatic tumor samples from autopsy can provide a comprehensive molecular portrait of advanced cancers that reflects changes in tumor cell populations and genomes over time [12, 13]. Here we performed research autopsy on a patient with widely metastatic IDCS. Genomic profiling of his tumors revealed somatic ultra-hypermutation and tumor heterogeneity in the form clonal diversity. Recently, Campbell *et al.* demonstrated a prevalence of ultra-hypermutation (TMB >100 mut/Mb) of 0.6% in a cohort of 78,452 adult cancers [4]. Therefore, the rare histologic classification of IDCS in this patient is accompanied by a rare ‘genotype’ of ultra-hypermutation.

Hypermutation in cancer may arise from intrinsic defects in DNA damage repair pathways or extrinsic mechanisms such as mutagenic exposure [4]. The majority of ultra-hypermutation detected by Campbell *et al.* was attributed to mutations in mismatch repair (MMR) genes and replication-associated DNA polymerases Polε or Polδ. While we did not detect genomic alterations involving MMR or Polε/Polδ to explain this patient’s ultra-hypermutated cancer, we did detect a truncal amplification of a region on chromosome 22q containing genes encoding the APOBEC3A-H (or A3 subfamily) cytidine deaminases. The deregulated expression and activity of A3A and A3B have been linked to cancer development through enhanced DNA hypermutation and unfaithful RNA editing [14–16]. Furthermore, we identified a truncal *RPA2* variant with a predicted (by DUET [17]) destabilizing missense mutation in the C-terminal winged helix domain important for protein-protein interaction that could have further contributed to defective DNA damage repair and hypermutation. Together with driver mutations in tumor suppressors *TP53* and *CDKN2A*, the above events could have produced ultra-hypermutation seen in this patient’s cancer.

Different models of mutation accumulation have been proposed for hypermutated cancers and could be classified as ‘steady’ versus ‘dynamic’ hypermutation [4]. In the steady model, mutations accumulate gradually due to continuous mutagenic exposure or germline MMR deficiencies, leading to hypermutation over time. The dynamic model, seen in cancers with Polε or Polδ mutations, is characterized by ‘bursts’ of mutations followed by rise in genome-wide TMB [4]. Through analyses of clonality and mutational signatures, we hypothesize a gradual mode of hypermutation consistent with the former model in our patient’s cancer. Genomic profiling of treatment-naïve tumor revealed high TMB at baseline that may have been the result of UV exposure, producing the driver mutations in *TP53* and *CDKN2A*. TMB analysis of his autopsy tumor samples demonstrated an increase of ∼20–37 additional mut/Mb that could be attributed to the truncal amplification of the A3 subfamily of cytidine deaminases, as indicated by the presence of APOBEC-specific Signature 2, and/or potentially therapy-induced mutations. Interestingly, the brain metastasis (T10) was the only autopsy tumor sample that had similar TMB as the treatment-naïve tumor (T1) while still retaining the APOBEC signature. A technical explanation underlying this finding may be due to lower TC contents in both samples affecting variant detection. Alternatively, this may reflect similar biology between the treatment-naïve tumor sample obtained from neck surgery and the brain metastasis; this similarity is demonstrated by the shorter genetic distance between ‘normal’ and T1 or T10 samples relative to other tumor samples in the NJ tree. Finally, the genetic dissimilarity between the brain metastasis and tumor samples from other organs also suggests the presence of unique genetic features of cancer cells with increased propensity for invasion of the central nervous system. Although high TMB has been established as a clinically useful biomarker of response to checkpoint blockade in multiple human cancers [18, 19], our patient developed progressive cancer while receiving adjuvant therapy with the PD-1 inhibitor nivolumab and had disease progression while being treated with dual CTLA-4/PD-1 blockade. Therefore, his case highlights the need for identification of additional biomarkers to predict clinical benefit for immunotherapy in patients with hypermutated cancers.

In summary, we present the first genomic characterization of metastatic IDCS, an extremely rare neoplasm of dendritic cell origin that lacks any standard therapy. This patient had metastatic IDCS characterized by ultra-hypermutation and clonal heterogeneity, likely through a combination of chronic mutagen exposure (UV), acquired defects in pathways important for DNA repair (*TP53, CDKN2A, RPA* mutations), and gain of genes that promote DNA hypermutation (*APOBEC3A-H*). His cancer was aggressive and refractory to multiple anti-cancer therapies including molecularly targeted agents and immunotherapies. In the future, it will be important to study additional patients with this and other rare cancers with hypermutation. Finally, the broader clinical implication of our results is that although patients with hypermutated cancer, originating from either somatic or germline genomic aberrations, are more likely to benefit from checkpoint inhibition, research is still needed to stratify these patients to maximize therapeutic efficacy and identify the different genetic determinants of primary or acquired resistance to immunotherapy.

## MATERIALS AND METHODS

### Research autopsy

The patient was consented to an IRB-approved clinical study for tumor profiling and body donation. At time of death, the patient’s next-of-kin (and/or hospice agency) notified members of the research team. The deceased was transported to the OSU Medical Center and a limited research autopsy was conducted within 3 hours after patient’s passing. Metastatic tumors and adjacent normal tissues were sampled and immediately archived as fresh frozen specimens in OCT medium. After conclusion of the autopsy, the deceased was transported back to the designated funeral home within 24 hours.

### Whole exome sequencing

We extracted genomic DNA from frozen tumors and normal (blood) samples and prepared sequencing libraries using an established protocol [20] that included enrichment with the xGEN^®^ Exome Research Panel v1.0 from Integrated DNA Technologies. Sequencing was performed on an Illumina HiSeq4000 at The Genomics Services Laboratory at Nationwide Children’s Hospital (Columbus, Ohio) and achieved a median depth of 100–286x.

### Alignment, variant calling, and annotation

Sequencing reads were aligned to hg19 using bwa [21] version 0.7.14. Deduplication was performed using Picard [22] version 2.3.0. Quality recalibration and local realignment around indels was performed with Picard and GATK [23] version 3.5. Somatic SNVs (single-nucleotide variations), somatic indels, and germline SNVs were called using VarScan2 [24] version 2.3.9. Somatic SNVs were filtered using bam-readcount as follows: minimum average base quality of variant-supporting reads 22, average variant distance to 3’ read end of 0.24, Fisher’s exact test *P* ≤ 0.05, maximum average sum of base quality mismatches 100, maximum average mismatches per base of variant-supporting reads 0.04, and minimum VF (variant fraction) 6%. Germline SNVs were filtered as above, except for Fisher’s exact test *P* ≤ 0.1 (as recommended by VarScan2 documentation). Somatic indels were filtered with Fisher’s exact test *P* ≤ 0.05 and VF ≥ 6%. In addition, a list of repetitive regions in hg19 was generated using the RepeatFinder tool included in MANTIS, modified to output all repeats of 1-mers to 5-mers spanning at least 4 bp. Indels falling within this list were excluded from further analysis.

Somatic SNVs and indels were annotated with ANNOVAR (revision #11f4bb, 2016-02-01) [25]. Mutational signatures from the COSMIC Mutational Signatures set [26] were called with deconstructSigs [27] version 1.8.0 with default settings and exome2genome trinucleotide frequency correction, run on R version 3.3.2. Microsatellite instability testing was performed using MANTIS version 1.0.3, run with three threads and otherwise default settings.

To compute a neighbor joining tree of per-sample phylogeny, we first generate a distance matrix $D\in {\mathbb{Z}}^{\left(N+1\right)\times \left(N+1\right)}$. For sample i ϵ1..*N*, define *S*_*i*_ as the set of somatic SNVs in that sample called as above. Define *S*_*N*+1_ as the empty set, representing germline (with no somatic SNVs by definition). We compute D as follows:

$$d_{ij}=\left|S_{i}\;\Delta \;S_{j}\right|$$

where △ is the set symmetric difference. Neighbor joining was performed over D using RapidNJ [28] version 2.3.2. The resulting tree was plotted with Interactive Tree of Life (iTOL) [29] version 4.2.3.

### Copy number variation

Copy number variations (CNVs) were called using FALCON version 0.2 with threshold 0.3, and the QC procedure provided with Canopy was run with default length and ΔCN settings. The read depth ratio for each sample was computed as the ratio of aligned reads in tumor to aligned reads in normal. CNVs called by FALCON were manually inspected to identify events with major copy number >2 or minor copy number < 0.5 in at least one tumor sample, and spanned at least 25% of a chromosome. For each of these CNVs, common breakpoints were estimated across all tumors. FALCON was then re-run in each sample with ${\hat{\tau }}_{\begin{array}{l} chr \end{array}}$ set to the nearest SNP and threshold 0.2, with manual rescue of CNVs (threshold ≥ 0.1 in all cases).

### Subclonal phylogenetic analysis

The reference read count, alternate allele count, and variant fractions of all nonsynonymous somatic variants called within each tumor sample were compiled. Somatic SNVs were further filtered with at least 100x coverage in all samples, minimum of 20 alt-supporting reads in at least one sample, not on a sex chromosome, and a DANN [30] predicted mutational impact score ≥ 0.96 (*n* = 893). This list of high-confidence mutations was downsampled to a set of 60 mutations with maximal diversity using a high correlation filter, implemented as follows: while |M|>n:

$$i\underset{\text{delete}\left(M_{i}\right)}{\leftarrow }\arg\;\max_{x}\left(\rho_{MxMy}\right);y>x;x,\mathrm{y\epsilon}1..\left|M\right|$$

where *M* is the set of mutations, with each mutation being a vector of its per-sample VFs, *n* is the desired final number of mutations (in this case, *n* = 60), and ρ is the correlation between VFs in each sample, defined as follows:

$$\rho_{\vec{a}\vec{b}}=\frac{Cov\left(\vec{a},\vec{b}\right)}{\sigma_{\vec{a}}\sigma_{\vec{b}}}$$

where Cov(*x*, *y*) is the covariance between elements of same-length vectors *x* and *y*, and σ_x_ is the population standard deviation of elements of vector *x*.

Canopy was then run with an in-house parallelized version of sample.cluster mode with cluster number from 2 to 9, 10 MCMC clustering runs, τ_K+1_ 0.05, number potential subclones from 3 to 9, 50 chains per subclone number, burnin 100, thinning parameter 5, simulation runs from 20000 to 50000, and writeskip 200. As FALCON does not generate standard deviations for regions with identical major and minor copy number, and Canopy does not support sparse *ԑ*_*M*_ or *ԑ*_*m*_ matrices, the Frobenius norm of non-NA values of the *ԑ*_*M*_ and *ԑ*_*m*_ matrices was used for Canopy. The optimal subclone number was selected by highest BIC (Bayesian Information Criterion) score.

Afterwards, the remaining somatic SNVs (not used for Canopy tree building) and indels were retroactively assigned to the resulting tree using a maximal likelihood model. For an arbitrary somatic mutation *v*, define *N* as the number of tumor samples, $\vec{r}\in {\mathbb{Z}}^{N}$ as the number of alternate-supporting reads for *v* in each sample, $\vec{x}\in {\mathbb{Z}}^{N}$ as the coverage of the variant position in each sample, and $\vec{p}\in {\mathbb{R}}^{N}$ a vector of expected variant fractions in each sample (derived from the tree as shown below). Given known $\vec{x}$ and $\vec{p}$, and modeling alternative read depth as jointly binomial across tumor samples, the probability and log-likelihood can be computed as follows:

$$P\left(\vec{r}|\vec{x},\vec{p}\right)=\prod_{i=1}^{N}\left(\begin{array}{c} x_{i}\\ r_{i} \end{array}\right)p_{i}^{r_{i}}{\left(1-p_{i}\right)}^{\left(x_{i}-r_{i}\right)}$$

$$\begin{array}{l} ℒ\left(v\in z\right)=\ln\left(P\left(\vec{r}|\vec{x},\vec{p}\right)\right)\\ =\sum_{i=1}^{N}\left[\sum_{j=r_{i}+1}^{x_{i}}\ln\left(j\right)-\sum_{j=1}^{x_{i}-r_{i}}\ln\left(j\right)+r_{i}\ln\left(p_{i}\right)+\left(x_{i}-r_{i}\right)\ln\left(1-p_{i}\right)\right] \end{array}$$

To compute $\vec{p}$ we utilize the phylogenic tree and clonal fractions reported by Canopy, along with its per-subclone major and minor copy number estimates. Define *Z* as the number of edges of the tree, and *K* as the number of tumor subclones + 1 (to account for normal cells). Given the tree, a matrix $T\in {\left\{0,1\right\}}^{Z\times K}$ representing the phylogenetic composition of each subclone can be constructed as follows (note that the tree root corresponds to germline):

$$T_{z,k}=\left\{ \begin{array}{l} 1,\;\text{if the path fromroot to subclone}\;k\;\text{contains edge}\;z\\ 0,\;\text{otherwise} \end{array}\right.,z=1..Z,k=1..K$$

Next, define $P\in {\mathbb{R}}^{K\times N}$ as the clonal fraction matrix provided by Canopy (where entries in the first column represent proportion of normal cells in each sample), *T* as the number of CNV regions used for Canopy, ${\tilde{C}}^{M}\in {\mathbb{Z}}^{\left(T\times K\right)}$ as the per-subclone major copy number matrix, and ${\tilde{C}}^{m}\in {\mathbb{Z}}^{\left(T\times K\right)}$ as the per-subclone minor copy number matrix. Any zero values within each column of *P* were set to 0.0005, with the remaining entries reduced equally to ensure that each column sums to 1. This is first used to compute $A\in {\mathbb{R}}^{\left(T\times N\right)}$ the total copy number of each CNV region in each sample (independent of any specific mutation), as follows:

$$A=\left({\tilde{C}}^{M}+{\tilde{C}}^{m}\right)P$$

Next, assume without loss of generality that *v* is within edge *z*. From Canopy, if *v* is within a CNV *t*, *t* is within edge *y*. We can calculate the copy number of *v* in each subclone, $\vec{b}\in {\mathbb{Z}}^{K}$, as follows:

$$\vec{b}={\left(T_{\mathrm{z*}}\circ C\right)}^{T},\mathrm{C=}\left\{ \begin{array}{l} {\tilde{C}}^{M}t_{*}\;,v\;\text{is on major allele of CNV}\;t,\text{and}\;y\;\text{is further from the root than}\;z\\ {\tilde{C}}^{m}t_{*}\;,v\;\text{is on major allele of CNV}\;t,\text{and}\;y\;\text{is further from the root than}\;z\\ {\left\{1\right\}}^{K},\;v\text{is not within a CNV},\text{or}\;z\;\text{is further from the root than}\;y \end{array}\right.$$

where ○ is the Hadamard product of matrices. This permits calculation of the copy number of *v* in each sample, under the assumptions that *v* ∈ *z* and *v* is on a major or minor allele, as follows:

$$\vec{s}=P^{T}\vec{b}$$

Computation of $\vec{p}$ for mutation *v* is now straightforward:

$$p_{i}=\frac{s_{i}}{a_{i}},i=1..N,a=\left\{ \begin{array}{l} At{*}^{T},v\;\text{is within CNV}\;t\\ {\left\{2\right\}}^{N},v\;\text{is within a CNV, and is on an autosome or chrX in a female}\\ {\left\{1\right\}}^{N},v\;\text{is on chrX or chrY in a male} \end{array}\right.$$

To perform the assignment, ℒ(v∈z) is computed as described above for all edges. If *v* is within a CNV region and the candidate edge is before the CNV, its possibilities of being on the major or minor allele are both considered. If the candidate edge is after the CNV, only the possibility of it occurring on one copy of the allele is considered, utilizing the simplifying assumptions (also made by Canopy) that each somatic mutation occurs only once and no back-mutations occur. If the candidate edge contains the CNV, all three above possibilities (major/minor/after) must be considered. To avoid numerical errors, if *p*_*i*_ = 0 and *r*_*i*_ ≠ 0 for a candidate assignment in any sample, that candidate assignment is discarded. The edge with highest log-likelihood is selected, with ties (such as can occur with deletions or on a different branch than a CNV) broken in favor of the edge furthest from the root node. This approach was implemented and run using Python 2.7.1, using scipy [31] 0.10.1.

## SUPPLEMENTARY MATERIALS FIGURES AND TABLES














