# Supplementary material for: Genomic characterization of metastatic ultra-hypermutated interdigitating dendritic cell sarcoma through rapid research autopsy
Source: Oncotarget. 2019 Jan 8;10(3):277–88. doi: 10.18632/oncotarget.26352 (PMC6349455; doi:10.18632/oncotarget.26352)
Supplement: Supplementary file 1 [file oncotarget-10-277-s001.pdf]

Genomic characterization of metastatic ultra-hypermutated interdigitating dendritic cell sarcoma through rapid research autopsy

SUPPLEMENTARY MATERIALS

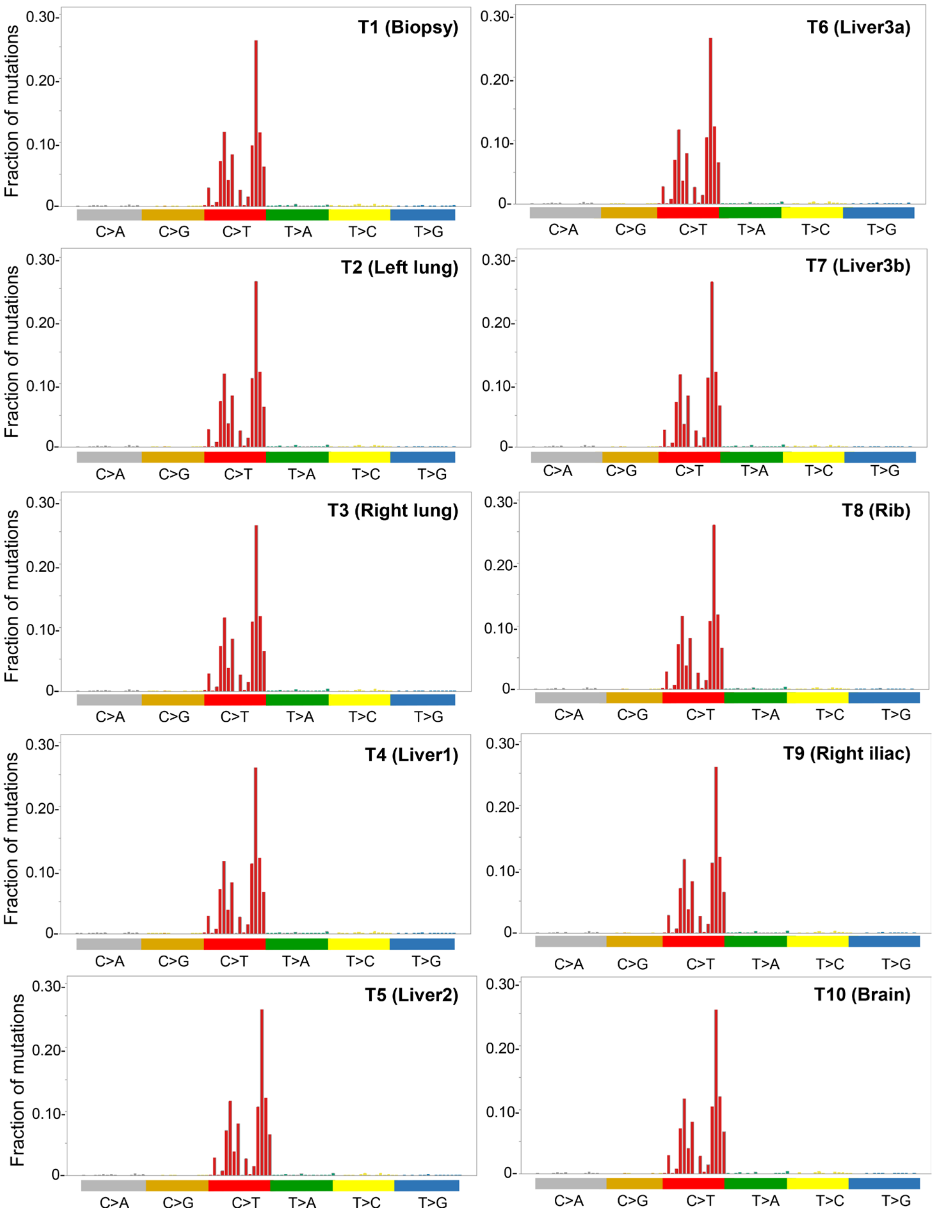

Supplementary Figure 1: Mutational spectra per tumor sample. Each lego plot portrays the distribution of mutations in each sample, given their trinucleotide context. The X-axis of lego plots contains 96 possible mutation types that result when the six classes of base substitutions (e.g. C>T) are placed in the trinucleotide context of flanking 5′ and 3′ bases. The Y-axis represents the fraction of base substitutions in each sample within each trinucleotide context.

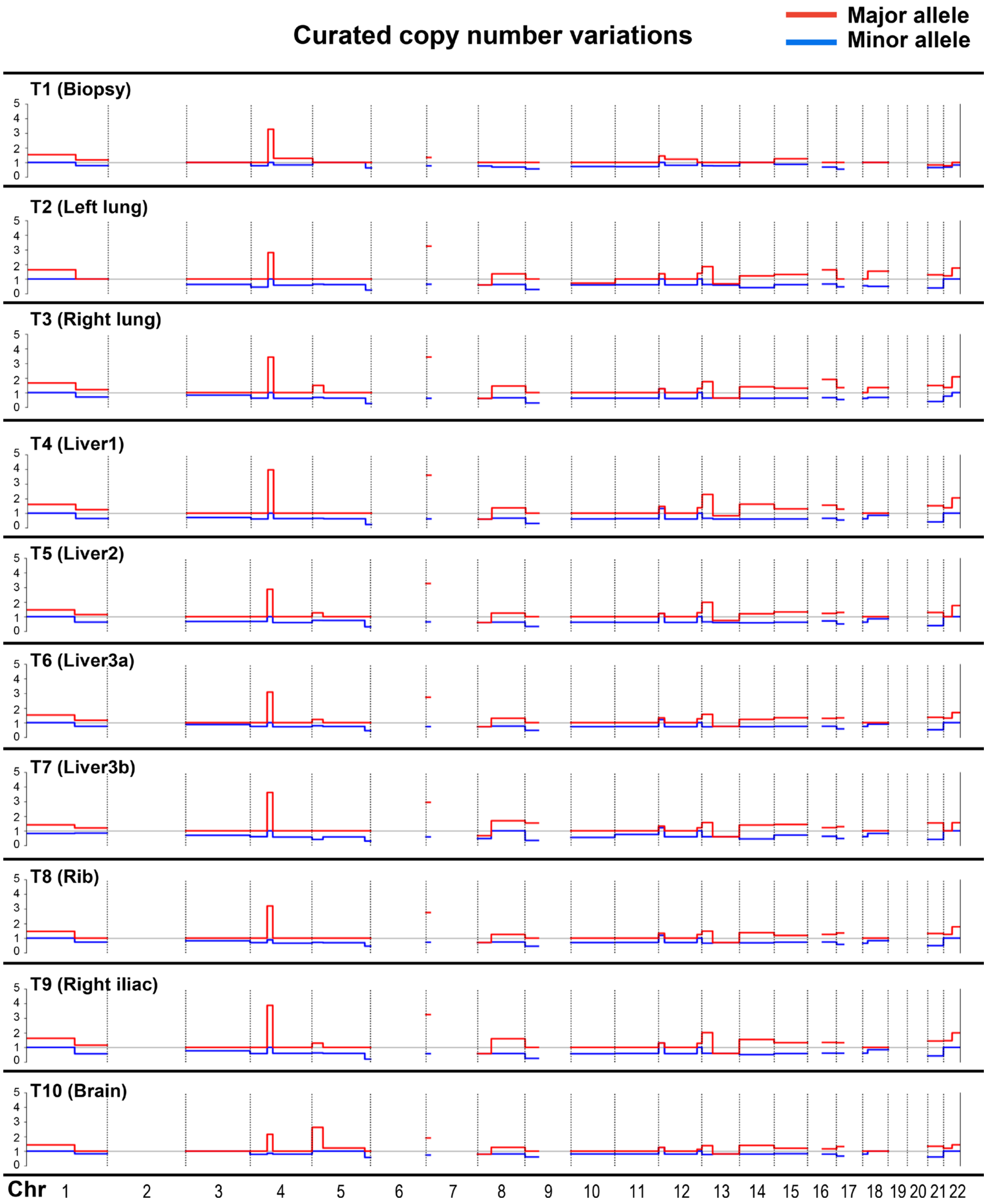

Supplementary Figure 2: Curated copy number variations per tumor sample. Copy number variations (CNVs) were called with FALCON, and manually curated on a per-sample basis. The copy number of the major allele (red) and minor allele (blue) is depicted for each tumor sample. Areas without a listed allele are copy-neutral.

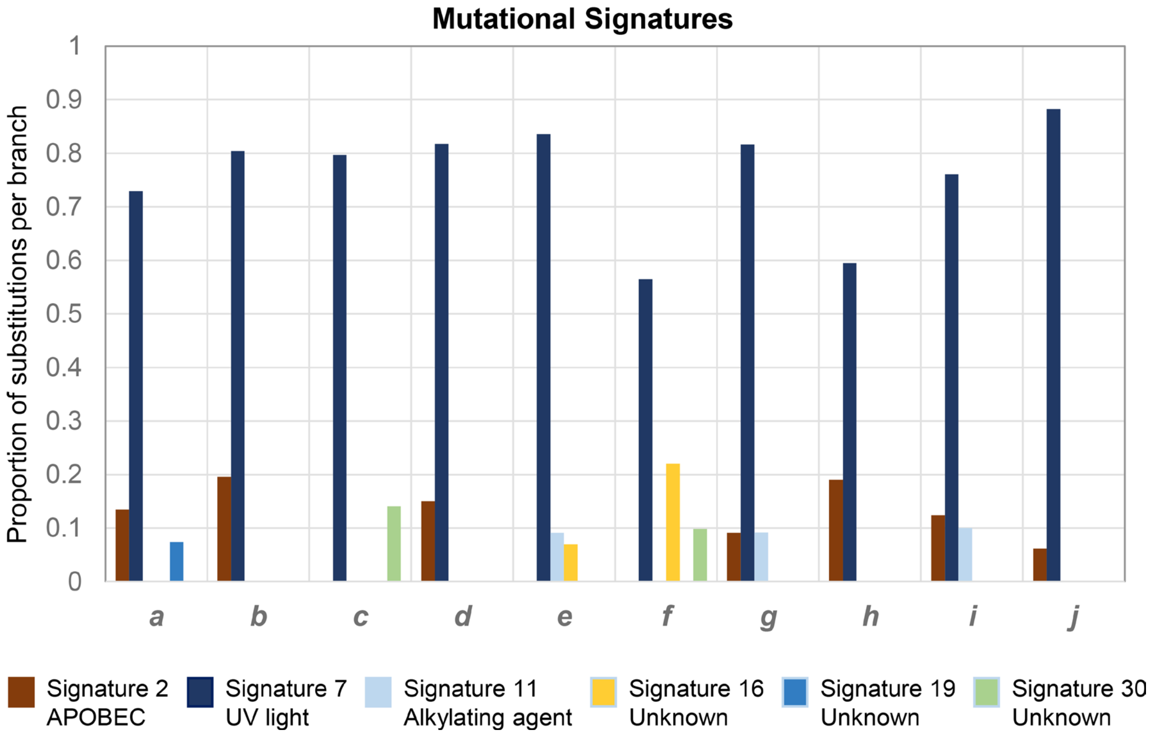

Supplementary Figure 3: Mutational signatures by phylogeny branch. Mutations in each branch of the phylogram (see Figure 3A) estimated by Canopy were called with deconstructSigs, utilizing the COSMIC Mutational Signatures set. Signature 7 (UV) was detected in every branch of the tumor's evolutionary history.

Supplementary Table 1: Annotated mutations by tumor sample. SNVs and indels passing quality control filters (see Supplementary Bioinformatics Methods; Alignment, Variant Calling, and Annotation) were annotated using ANNOVAR. See Supplementary_Table_1

Supplementary Table 2: Mutational signatures per tumor and per phylogeny branch. Mutations in each tumor and each branch were called with deconstructSigs, utilizing the COSMIC Mutational Signatures set. See Supplementary_Table_2

Supplementary Table 3: Raw FALCON CNV output. Allele-specific CNVs were called using FALCON with the QC procedure provided with Canopy. Output prior to manual curation is provided in this file. See Supplementary_Table_3

Supplementary Table 4: Curated CNVs by tumor sample. For each of the 29 CNV-affected regions identified and manually curated, the major and minor copy number and standard deviations are listed. These values were determined through execution of FALCON with manual selection of SNVs for segmentation. See Supplementary_Table_4

Supplementary Table 5: Estimated clonal composition of all tumor samples

	T1	T2	T3	T4	T5	T6	T7	T8	T9	T10
normal	0.414	0.217	0.007	0.001	0.078	0.227	0.133	0.227	0.007	0.397
clone1	0.153	0.065	0.305	0.341	0.279	0.266	0.231	0.251	0.294	0.194
clone2	0.095	0.256	0.242	0.208	0.21	0.175	0.157	0.157	0.222	0.103
clone3	0.117	0	0.001	0.001	0.018	0.001	0.005	0.006	0.005	0.061
clone4	0.026	0.078	0.065	0.065	0.064	0.063	0.091	0.067	0.074	0.068
clone5	0.187	0.305	0.291	0.326	0.276	0.222	0.285	0.23	0.346	0.176
clone6	0.008	0.079	0.089	0.058	0.075	0.046	0.098	0.062	0.052	0.001

For each of 10 tumor samples, the estimated fraction of normal and tumor cells from each clone is listed.

Supplementary Table 6: Annotated mutations by phylogeny branch. SNVs and indels are listed for each branch a–j of the phylogram (see Figure 3A). Mutations in bold were used by Canopy to build the tree, and the remaining mutations were retroactively assigned to the tree (see Supplementary Bioinformatics Methods; Phylogenetic Analysis). See Supplementary_Table_6

Supplementary Table 7: Curated CNVs by phylogeny branch. For each of the 29 CNV-affected regions identified and manually curated, the per-subclone major and minor copy number as estimated by Canopy is listed. See Supplementary_Table_7
